# Pollen-Inspired Photonic Barcodes with Prickly Surface for Multiplex Exosome Capturing and Screening

**DOI:** 10.34133/2022/9809538

**Published:** 2022-08-31

**Authors:** Ning Li, Feika Bian, Xiaowei Wei, Lijun Cai, Hongcheng Gu, Yuanjin Zhao, Luoran Shang

**Affiliations:** ^1^Department of Rheumatology and Immunology, Institute of Translational Medicine, Nanjing Drum Tower Hospital, School of Biological Science and Medical Engineering, Southeast University, Nanjing 210096, China; ^2^Oujiang Laboratory (Zhejiang Lab for Regenerative Medicine, Vision, And Brain Health), Wenzhou Institute, University of Chinese Academy of Sciences, Wenzhou, Zhejiang 325001, China; ^3^Shanghai Xuhui Central Hospital, Zhongshan-Xuhui Hospital, And the Shanghai Key Laboratory of Medical Epigenetics, The International Co-Laboratory of Medical Epigenetics and Metabolism (Ministry of Science and Technology), Institutes of Biomedical Sciences, Fudan University, Shanghai, China

## Abstract

Exosomes, which play an important role in intercellular communication, are closely related to the pathogenesis of disease. However, their effective capture and multiplex screening are still challenging. Here, inspired by the unique structure of pollens, we present novel photonic crystal (PhC) barcodes with prickly surface by hydrothermal synthesis for multiplex exosome capturing and screening. These pollen-inspired PhC barcodes are imparted with extremely high specific surface area and excellent prickly surface nanostructures, which can improve the capture rate and detection sensitivity of exosomes. As the internal periodic structures are kept during the hydrothermal synthesis process, the pollen-inspired PhC barcodes exhibit obvious and stable structural colors for identification, which enables multiplex detection of exosomes. Thus, the pollen-inspired PhC barcodes can not only effectively capture and enrich cancer-related exosomes but also support multiplex screening of exosomes with high sensitivity. These features make the prickly PhC barcodes ideal for the analysis of exosomes in medical diagnosis.

## 1. Introduction

Exosomes, which are a class of extracellular vesicles secreted by almost all cells, have come to the forefront in biomedical field during the last decade [1–3]. Since they carry metabolism-related molecules including proteins and RNA [4, 5], exosomes are recognized as important mediators for regulation of cell biological behaviors as well as potential biomarkers for the diagnosis of many diseases [6–9]. Attracted by this, researchers have paid much attention in developing efficient approaches to capture and enrich exosomes [10–13], which are two requisite operations for analysis of exosomes. As a result, many methods have been proposed, including size-exclusion chromatography, and immunological capture. Particularly, some micro- and nanotechnologies, such as antibody-modified magnetic nanobeads or microfluidic chips [14, 15], have been converted to commercial exosome kits due to their advantages of simple operation, low cost, and high sensitivity. Although with many successes, most of these existing carriers possess simple surface structures, which would hinder them from efficient capture of exosomes. In addition, it is alluring yet unmet to realize multiplex capturing and screening of exosomes. Thus, new stratagem for effectively capturing and screening of exosomes is still anticipated.

**Figure 1:**
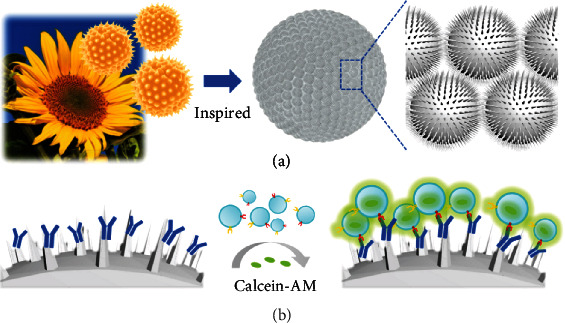
Schematic illustration of the design and application of the pollen-inspired PhC barcodes. (a) Micromorphology of pollens from sunflower and pollen-inspired photonic barcodes with prickly surface. (b) The pollen-inspired PhC barcodes with prickly surface could be modified with antibodies, and then exosomes could be captured on the barcodes efficiently due to the specific recognition. Calcein-AM was employed as a fluorescent indicator of exosomes.

Here, inspired by the unique structure of pollens, we present novel photonic crystal (PhC) barcodes with prickly surface for multiplex capturing and screening of exosomes, as schemed in Figure 1. Pollens are generally microparticles, some of which exhibit a variety of projections (such as granular, rod-shaped, striate, spiny, etc.) on their surface. Benefiting from the special surface structure, pollens are imparted with high specific surface area and strong adsorbability, attracting much attention from diverse fields [16–19]. Meanwhile, PhC barcodes have been serving as powerful tools for multiplex assays because of their remarkable characteristic reflection peaks resulting from periodic ordered nanostructures [20–23]. As their encoding elements depend on physical structures, PhC barcodes are able to get rid of photobleaching and fluorescent background, exhibiting stable encoding property [24, 25]. These features make the PhC barcodes highly promising for many biomedical applications that multiplexing is needed [26, 27]. Therefore, it is convinced that the integration of pollen-inspired nanostructures onto the surface of PhC barcodes can open up a new multiplexing platform for effectively capturing and screening exosomes.

In this paper, the pollen-inspired PhC barcodes with prickly surfaces were achieved by simple hydrothermal treatment of the barcode particles [28]. During this process, the prickly nanostructures on barcode surfaces could be controllably adjusted through changing the reactants concentration and reaction time. Benefiting from the prickly structures, the resultant pollen-inspired PhC barcodes showed an extremely high specific surface area, which allowed efficient probe modifications and improved sensitivity in exosome capturing. As the hydrothermal synthesis process had little influence on the internal periodic structure, the prickly PhC barcodes still displayed bright structural colors for identification. Thus, by modifying different probes on the corresponding prickly PhC barcodes, multiplex enriching and screening of cancer-related exosomes can be realized with high sensitivity. These results indicated that the proposed pollen-inspired PhC barcodes with prickly surface have great potential in biomedical research and medical diagnosis.

## 2. Results

**Figure 2:**
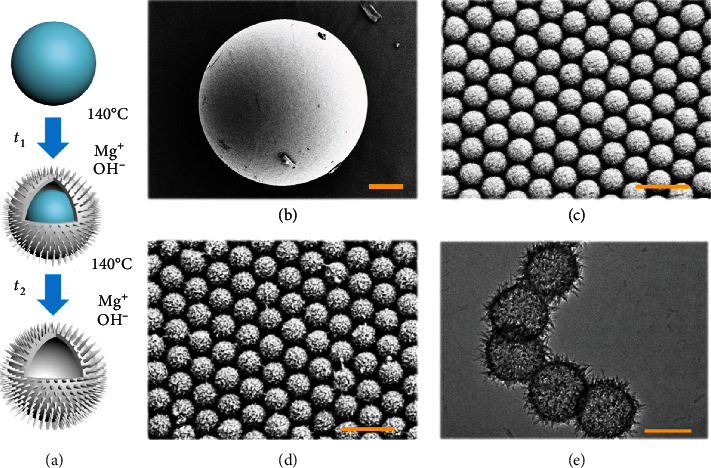
SEM and TEM images of the pollen-inspired PhC barcodes. (a) Schematic illustration of processing of nanoparticle on the pollen-inspired PhC barcodes. (b) SEM image of a pollen-inspired PhC barcode particle. (c) SEM image of the surface of the PhC template. (d) SEM image of the surface of the pollen-inspired PhC barcode. (e) TEM image of silicate nanoparticles. Scale bars are 50 *μ*m in (b), 500 nm in (c, d), and 200 nm in (e).

In a typical experiment, pollen-inspired PhC barcodes were generated by hydrothermal technique employing PhC particles as sacrificial templates. Silica nanoparticles first self-assembled in microfluidic droplets, and then the PhC templates with bright structural colors were developed. Subsequently, the PhC templates were incubated in ammonia solution at high temperature for separating out the silicate ions. Then, the silicate ions could react with metal ions to form silicate, which were preferentially generated on the surface of silica nanoparticles. As the reaction progressed, the silicate shell formed and grew gradually, resulting in surface prickly microspheres with a pollen-like structure, as schemed in Figure 2(a). Scanning electron microscope (SEM) was employed to confirm their structures during the fabrication process (Figures 2(b)–2(d)). The PhC templates' nanoparticles were arranged firmly in an ordered hexagonal alignment (Figure 2(c)). Notably, after the hydrothermal process, spines appeared on the surface of the nanoparticles, while the nanoparticles still remained a tight hexagonal arrangement (Figure 2(d)). The nanoparticles with interior hollow and external nanoneedle structure were observed by transmission electron microscope (TEM) (Figure 2(e)). Besides, the average particle size of these barcodes was 266.8 *μ*m (Figure S1).

**Figure 3:**
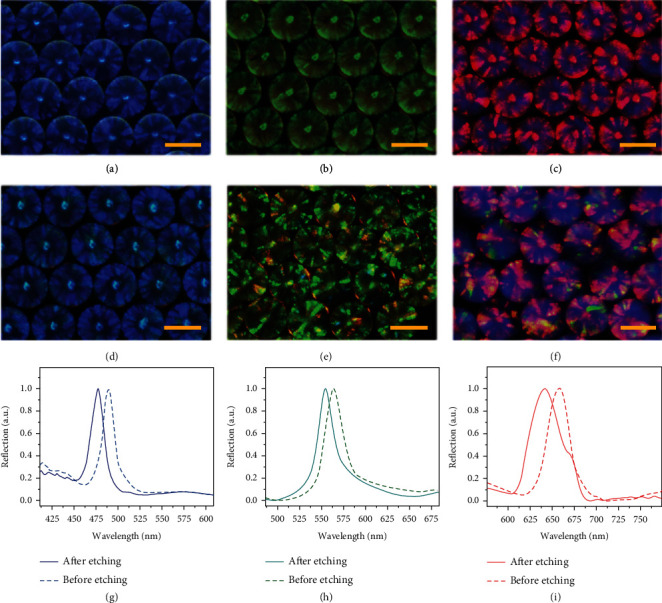
Optical characterization of the pollen-inspired PhC barcodes. (a)–(c) Reflection images of three kinds of PhC templates. (d)–(f) Reflection images of three kinds of pollen-inspired PhC barcodes. (g)–(i) Reflection spectra of these three kinds of pollen-inspired PhC barcodes before and after etching. Scale bars are 200 *μ*m in (a)–(f).

Due to the regular nanostructures, the pollen-inspired PhC barcodes possessed photonic band gap (PBG) characteristics, which imparted them with distinct structural colors and prominent reflection peaks. Bragg's equation can be used to the position of the characteristic reflection peak at normal incidence.

(1) $$\begin{array}{c} \lambda =1.633\text{d}n_{\text{average}}, \end{array}$$

where *n*_averag*e*_ refers to the pollen-inspired PhC barcodes' average refractive index, *d* is on behalf of the distance between two adjacent nanoparticles in the barcodes, and *λ* represents the peak wavelength. For a fixed composition, *n*_average_ is essentially the same under certain conditions. Therefore, to create multicolored barcodes with varied characteristic reflection peaks, the center distance *d* can be adjusted using nanoparticles of various sizes. Compared with the PhC templates (Figures 3(a)–3(c)), it was found that the reflection peak of the silicate pollen-inspired PhC barcodes appeared tiny changes (Figures 3(d)–3(f)). The position of the reflection peak was slightly blue-shifted after being etched, according to spectrometer measurements (Figure 3(g)–3(i)). By comparing the SEM images, we could find that the distance between two adjacent nanoparticles is essentially unaltered after being etched (Figures 2(c) and 2(d)). Thus, the blue shift of the reflection peak could be attributed to the change of the average refractive index (*n*_average_) caused by the variation of internal structure and composition of the particles. These results showed that the surface spines had almost no effect on the structural colors of the pollen-inspired PhC barcodes, indicating that the pollen-inspired PhC barcodes still exhibited stable coding capacity for multiplex biological detection.

**Figure 4:**
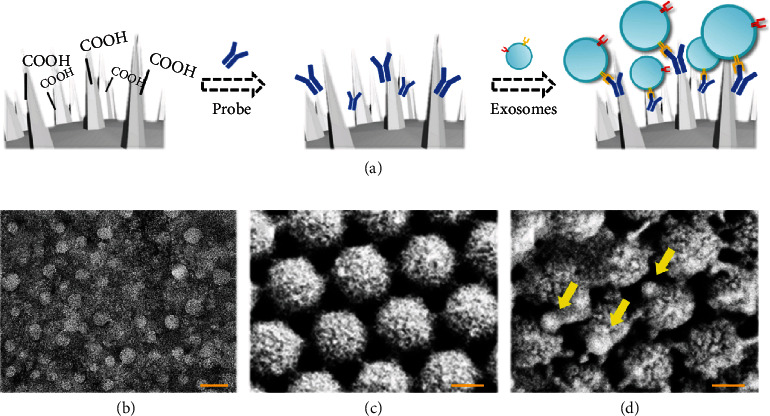
The exosome capture ability of the pollen-inspired PhC barcodes. (a) Scheme of process of modifying specific antibody probes on the surface of the pollen-inspired PhC barcodes. (b) TEM image of the exosomes morphology. (c) SEM image of the surface of pollen-inspired PhC barcodes before exosomes capturing. (d) SEM image of exosomes capturing by probe modified pollen-inspired PhC barcodes; the arrows indicate exosomes being captured. Scale bars are 100 nm in (b)–(d).

Then, the pollen-inspired PhC barcodes were modified with specific antibody probes to capture exosomes. Since the pollen-inspired PhC barcodes contained hydroxyl groups, the microspheres were firstly modified with amino groups by immersion in the ethanol solution of APTES. For the carboxylation of these barcodes, they were soaked in a succinic anhydride solution. Then, antibodies with the amino group were modified into these barcodes by covalent coupling (Figure 4(a)). The Fourier transform infrared spectroscopy (FTIR) analysis showed that the barcodes were successfully modified by carboxyl groups (Figure S2). In order to verify their ability to capture exosomes, the modified pollen-inspired PhC barcodes were incubated with exosomes obtained by ultracentrifugation (Figure 4(b)). As shown in Figures 4(c) and 4(d), after capturing, a large number of exosomes were observed on the modified barcodes surface. These results indicated that the surface of the pollen-inspired PhC barcodes had been successfully modified with the antibody probes, exhibiting remarkable ability for efficient capture of exosomes.

**Figure 5:**
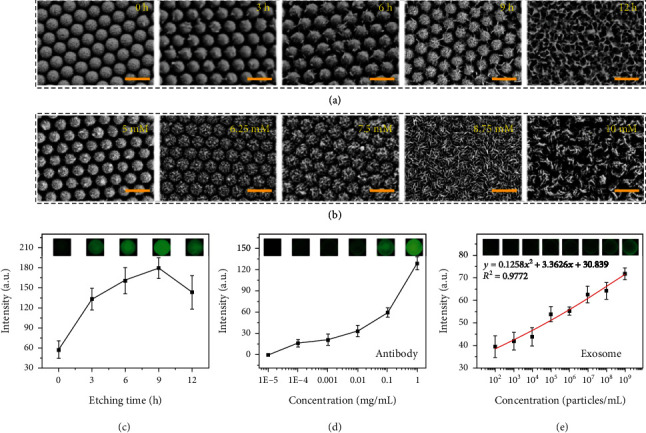
Optimization of the reaction conditions. (a) SEM images of the surface of pollen-inspired PhC barcodes treated for different corrosion times. (b) SEM images of the surface of pollen-inspired PhC barcodes treated at different ion concentrations. (c) Plot of the BSA-FITC fluorescence intensity of the pollen-inspired PhC barcodes (reflecting the antibody loading ability) as a function of the corrosion time. (d) Plot of the BSA-FITC fluorescence intensity of the pollen-inspired PhC barcodes as a function of the antibody concentration. (e) Plot of the calcein-AM fluorescence intensity (reflecting the capture ability) of the exosomes captured by pollen-inspired PhC barcodes as a function of the exosome concentration. Scale bars are 400 nm in (a, b).

The capture ability of exosomes is closely related to the specific surface area of the pollen-inspired PhC barcodes. In order to further improve the capture performance of the pollen-inspired PhC barcodes for exosomes, we explored the capture efficacy of the pollen-inspired PhC barcodes as a function of the corrosion time and ion concentration. When the magnesium ion concentration was 5 mM, the prickly structure on the pollen-inspired PhC barcodes surface became more obvious with the prolongation of corrosion time, and the 9 h group showed the best performance (Figure 5(a)). However, when the corrosion time reached 12 h, no prickly structure could be seen on the surface due to excessive corrosion. Correspondingly, the ability of pollen-inspired PhC to carry antibodies also increased with the increase of spines but decreased when the corrosion was excessive (Figure 5(c)). This was related to the change of specific surface area of the pollen-inspired PhC barcodes. As shown in Figure 5(b) and Figure S3, when the corrosion time was 9 h, with the increase of the magnesium ion concentration, the prickly structure on the surface of pollen-inspired PhC barcodes became more obvious. However, when the magnesium ion concentration reached over 8.75 mM, the irregular surface with excessive corrosion also appeared. Similarly, the ability of pollen-inspired PhC barcodes to carry antibodies increased with specific surface area and decreased when the corrosion was excessive (Figure S4). As we continue to increase the concentration of magnesium ions and ammonia, the mechanical strength of the barcodes would decrease, and the barcodes would fracture (Figure S5). Therefore, the magnesium ion concentration of 7.5 mM and the reaction time of 9 h were selected as the optimal reaction conditions. In order to optimize the working concentration of the antibody, the pollen-inspired PhC barcodes were modified with different concentrations of BSA-FITC for simulation, ranging from 0.01 *μ*g/mL to the maximum working concentration of the antibody 1 mg/mL. As shown in Figure 5(d), we found that the fluorescence intensity increased with the increase of antibody concentration, and it did not reach the plateau even at the maximum working concentration. Therefore, in our subsequent experiments, the maximum concentration of antibody was used as the working concentration. In order to explore the response of the pollen-inspired PhC barcodes to exosome concentration, different concentrations of exosome and antibody-modified pollen-inspired PhC barcodes were coincubated for 8 h. It was found that the response of pollen-inspired PhC barcodes to exosome could be as low as 100 particles/mL (Figure 5(e)).

**Figure 6:**
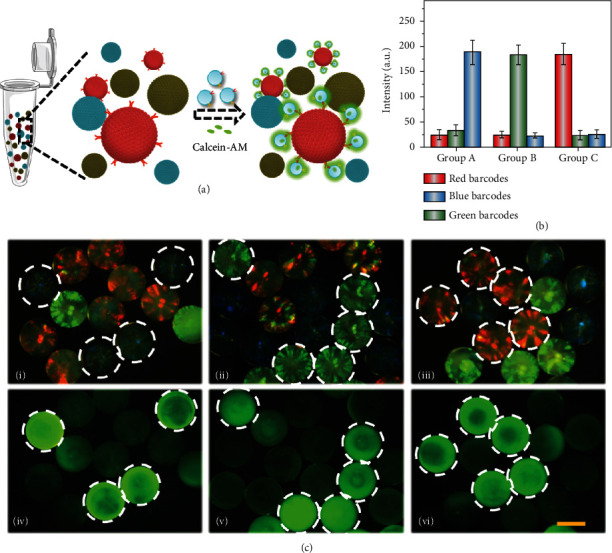
Multiple screening of cancer-related exosomes. (a) The specificity diagram of pollen-inspired PhC barcodes for cancer detection. (b) The fluorescence statistics of three different groups after incubating with bladder cancer exosomes. (c) Optical microscope images (i-iii) and fluorescence images (iv-vi) of three kinds of barcodes after incubating with bladder cancer exosomes. Scale bars are 200 *μ*m in (c).

The optimized experimental parameters were set and were used for the capture of exosomes secreted by cancer cells. As shown in Figure 6, pollen-inspired PhC barcodes with characteristic reflection peak of 470 nm, 550 nm, and 630 nm, appearing as blue, green, and red, respectively, were employed for the multiplex detention of exosomes. First, a series of barcodes with different corrosion time were prepared to investigate the nonspecific capture ability of exosomes (Figure S7). Results showed that barcodes treated for 6 h exhibited the best performance, which had obvious prickly surface and low nonspecific background at the same time. Then, we divided them into three groups, namely, blank control, bovine albumin (BSA) blocking, and modified with CD9/CD63/CD81 antibodies, as shown in Figure S8. Due to the special surface structure, the three groups incubated with exosomes showed different fluorescence after staining with calcein. It was found that the antibody-modified barcodes exhibited the brightest fluorescence, while barcodes of the blank groups had weak fluorescence and the BSA blocking groups had almost no fluorescence. This indicated that these barcodes could achieve screening of exosomes by coupling specific antibodies. For further applications, we prepared different barcodes of bladder cancer specific probes (human bladder tumor antigen (BTA) antibody, nuclear matrix protein 22 (NMP22) antibody, fibrocyte-derived protein (FDP) antibody), as shown in Figures 6(b) and 6(c). The first column selected blue barcodes coupling BTA probes and mixed with two other blank barcodes. After co-incubation with bladder cancer exosomes, the fluorescence signal of calcein in exosomes could be observed in blue barcodes (Figure 6(c) i, iv). Other combinations of NMP22/FDP probes coupled with green or red barcodes showed similar results (Figure 6(c) ii-iii, v-vi). These results suggested that the pollen-inspired PhC barcodes have potential applications in the multiplex screening of cancer.

## 3. Discussion

In conclusion, we have developed a novel pollen-inspired PhC barcode with prickly surface for both exosomes capture and multiplex detection. Pollen-inspired PhC barcodes were imparted with protruding spiny structure after hydrothermal synthesis treatment. Such structure not only had little influence on the excellent structure color of photonic crystals but also greatly increased the specific surface area of the microspheres and enhanced their adsorption capacity. Our experiments confirmed that pollen-inspired PhC barcodes combined with exosome-specific antibodies could perform multiplex screening of exosomes. In addition, because of the stable structural color, the barcodes could be conveniently distinguished by reflection peak. By using different barcodes with specific probes, different tumor markers on exosomes could be detected simultaneously. These results indicated that the present PhC barcodes with prickly surface could not only capture exosomes but also realize multiplex screening of exosomes. Therefore, the pollen-inspired PhC barcodes were expected to open a new chapter in the field of exosome capturing and analysis.

## 4. Materials and Methods

### 4.1. Materials

Sinopharm Chemical Reagent Co., Ltd. provided ammonium hydroxide (NH3·H2O) and ethanol (Shanghai, China). Aladdin Reagent Co., Ltd. offered succinic anhydride, N-hydroxysuccinimide (NHS), dimethyl sulfoxide (DMSO), and 1-ethyl-3-(3-dimethylaminopropyl) carbodiimide (EDC), 4-morpholineethanesulfonic acid hydrate (MES), and (3-aminopropyl) triethoxysilane (APTES). Magnesium chloride and ammonium chloride were purchased from Macklin. Dulbecco's Modified Eagle Medium (DMEM) was obtained from Gibco (USA). CD9, CD63, CD81, BTA, NMP22, and FDP antibodies were obtained from Abcam (China, Shanghai). Silica colloidal solutions were self-prepared. All reagents were applied as received with no further purification.

### 4.2. Preparation of PhC Templates

Based on our previous work, the PhC templates were obtained by assembling silica nanoparticles in microfluidic droplets [29]. The aqueous suspension containing silica nanoparticles was the dispersed phase. The continuous phase was silicon oil (Shinetsu, 50 CS, Japan). Monodisperse droplets were formed in a microfluidic device with a coflow geometry under shear force and surface tension. The droplets were then collected by a container of silicone oil with a high viscosity (500 CS). After 12 h of evaporation at 75°C, the droplets consolidated into solid beads. Then, hexane was used to clean the particles several times for remove silicone oil. At last, PhC templates were calcined to a stable structure at 800°C for 4 h.

### 4.3. Preparation of Pollen-Inspired PhC Barcodes

To prepare pollen-inspired PhC barcodes, 0.75 mmol magnesium chloride, 10 mmol ammonium chloride, and 1 mL ammonia were mixed in 30 mL deionized water to get magnesium chloride mixed reserve solution at room temperature. The PhC templates (0.1 g) were dispersed in 6.67 mL deionized water and then mixed with 10× diluted magnesium chloride mixed reserve solution (10 mL). The above solution was transferred into a reaction kettle for 6 h at 140°C. The products were collected and cleaned with deionized water and ethanol. Metal salts such as copper nitrate and nickel chloride could also be used for first step.

### 4.4. Surface Modification of Pollen-Inspired PhC Barcodes

The surface of the barcodes was aminated in ethanol solution containing 5% APTES for 4 h and washed with ethanol and deionized water. The barcodes were then soaked in a DMSO solution with 10 mg/mL succinic anhydride for 12 h for carboxylation. After washed with PBS buffer for three times, the treated barcodes were immersed in 1 mL of MES buffer at pH 6.0, with NHS and EDC to activate carboxyl groups. After that, the pollen-inspired PhC barcodes with carboxylated surface were placed in a PBS buffer solution of the amino-modified probes for 12 h at 37°C, so as to realize covalent coupling and fixation of the protein on the pollen-inspired PhC barcodes' surface.

### 4.5. Cell Culture

The frozen bladder cancer T24 cell lines were resuscitated in a 37°C water bath. Then, the cells were cultured in the DMEM (Gibco) in an incubator at 37°C with 5% CO_2_ for 2-3 days.

### 4.6. Preparation of UC-Purified Exosomes

The exosomes used were all purified by ultraspeed centrifuge (UC). Before preparing UC-purified exosomes, T24 cells were firstly cultured in medium for 2 weeks. The culture medium was then collected and centrifuged through a series of centrifugation steps at 3,000 rpm for 10 min and 10,000 rpm for 10 min to remove the cells, large vesicles, and cell debris, and then ultrafiltration membrane was used for further filtration. The supernatant obtained was centrifuged at an overspeed of 100,000 rpm for 90 min at 4°C, and the supernatant was discarded. The precipitation obtained was exosomes. Next, exosomes were rinsed with PBS, collected into one tube, and centrifuged at a speed of 100,000 rpm for 90 min again, and exosomes were recovered. The precipitation was then suspended with 100 *μ*L PBS and stored at 4°C.

### 4.7. Capturing and Screening of Exosomes

After being modified successfully, the pollen-inspired PhC barcodes were coincubated with exosomes at 37°C. After the capturing process, the barcodes were washed with PBS buffer for several times to eliminate the nonspecific adsorption on the surface of the microspheres. Then, 0.1% (v/v) calcein-AM was added into the reaction system after capturing, and the barcodes were incubated for 0.5 h for fluorescence detection.

### 4.8. Characterization

A field emission scanning electron microscope (FESEM, Zeiss, Ultra Plus) and a transmission electron microscope (TEM, JEOL, JEM-2100) were employed to characterize the structures of the barcodes. A CCD camera (Olympus, DP30BW) equipped on a stereoscopic microscope (Jiang Nan) or fluorescence microscope (Olympus, BX51) was used to collect optical and fluorescence images of the barcodes. The reflection peak of the pollen-inspired PhC barcodes was recorded by a fiber optic spectrometer (Ocean Optics, USB2000-FLG) equipped on the same microscope.

## Data Availability

All data are contained in the manuscript text and supplementary materials.

## Supplementary Materials

Supplementary Materials Figure S1: the particle size distribution map of pollen-inspired PhC barcodes. The average particle size is 266.8 *μ*m; standard deviation is 1.88. Figure S2: Fourier-transform infrared spectroscopy (FTIR) analysis of the pollen-inspired PhC barcodes before and after carboxyl group modification. Figure S3: SEM images of the surface of the pollen-inspired PhC barcodes treated with different ion concentrations. Scale bars are 200 nm Figure S4: (a)–(h) fluorescence images of pollen-inspired PhC barcodes with different ion concentration after BSA-FITC loading. (i) Optimization curve of the relationship between ion concentration and pollen-inspired PhC barcodes antibody loading ability. Scale bars are 200 *μ*m. Figure S5: optical characterization of the fragmented PhC barcodes due to excessive corrosion. Scale bars are 200 *μ*m. Figure S6: nanoparticle tracking analysis (NTA) curve of exosomes sample after diluted 100 times. The sample concentration was “4.26 × 10^8^” particles/mL. Figure S7: (a)–(e) fluorescence images after blank pollen-inspired PhC barcodes with different etching times (0 h, 3 h, 6 h, 9 h, 12 h) after exosomes capturing. (f) Curve of the relationship between etching time and fluorescence intensity of blank pollen-inspired PhC barcodes after exosomes capturing. Scale bars are 200 *μ*m. Figure S8: the fluorescence images of three groups: (a) blank control, (b) BSA blocking, and (c) modified with CD9/CD63/CD81 antibodies. Scar bar is 100 *μ*m.
