# Supplementary material for: Pollen-Inspired Photonic Barcodes with Prickly Surface for Multiplex Exosome Capturing and Screening
Source: Research (Wash D C). 2022 Aug 31;2022:9809538. doi: 10.34133/2022/9809538 (PMC9470204; doi:10.34133/2022/9809538)
Supplement: Supplementary Materials — Figure S1: the particle size distribution map of pollen-inspired PhC barcodes. The average particle size is 266.8 μm; standard deviation is 1.88. Figure S2: Fourier-transform infrared spectroscopy (FTIR) analysis of the pollen-inspired PhC barcodes before and after carboxyl group modification. Figure S3: SEM images of the surface of the pollen-inspired PhC barcodes treated with different ion concentrations. Scale bars are 200 nm Figure S4: (a)–(h) fluorescence images of pollen-inspired PhC barcodes with different ion concentration after BSA-FITC loading. (i) Optimization curve of the relationship between ion concentration and pollen-inspired PhC barcodes antibody loading ability. Scale bars are 200 μm. Figure S5: optical characterization of the fragmented PhC barcodes due to excessive corrosion. Scale bars are 200 μm. Figure S6: nanoparticle tracking analysis (NTA) curve of exosomes sample after diluted 100 times. The sample concentration was “4.26 × 10^8^” particles/mL. Figure S7: (a)–(e) fluorescence images after blank pollen-inspired PhC barcodes with different etching times (0 h, 3 h, 6 h, 9 h, 12 h) after exosomes capturing. (f) Curve of the relationship between etching time and fluorescence intensity of blank pollen-inspired PhC barcodes after exosomes capturing. Scale bars are 200 μm. Figure S8: the fluorescence images of three groups: (a) blank control, (b) BSA blocking, and (c) modified with CD9/CD63/CD81 antibodies. Scar bar is 100 μm. [file 9809538.f1.docx]

Supporting Information

*Pollen-inspired Photonic Barcodes with Prickly Surface for Multiplex Exosome Capturing and Screening*

*Ning Li^1^, Feika Bian^1^, Xiaowei Wei^1^, Lijun Cai^1^,* *Hongcheng Gu ^1,^*, Yuanjin Zhao^1,3,^*, Luoran Shang^1,2,^**

1. Department of Rheumatology and Immunology, Institute of Translational Medicine, Nanjing Drum Tower Hospital, School of Biological Science and Medical Engineering, Southeast University, Nanjing 210096, China.

2. Shanghai Xuhui Central Hospital, Zhongshan-Xuhui Hospital, and the Shanghai Key Laboratory of Medical Epigenetics, the International Co-laboratory of Medical Epigenetics and Metabolism (Ministry of Science and Technology), Institutes of Biomedical Sciences, Fudan University, Shanghai, China

3. Oujiang Laboratory (Zhejiang Lab for Regenerative Medicine, Vision and Brain Health), Wenzhou Institute, University of Chinese Academy of Sciences, Wenzhou, Zhejiang 325001, China

Correspondence should be addressed to Hongcheng Gu; [hcgu@seu.edu.cn](mailto:hcgu@seu.edu.cn) , Yuanjin Zhao; [yjzhao@seu.edu.cn](mailto:yjzhao@seu.edu.cn) and Luoran Shang; [luoranshang@fudan.edu.cn](mailto:luoranshang@fudan.edu.cn)

**Supplementary Figures**

**Figure S1.**

**
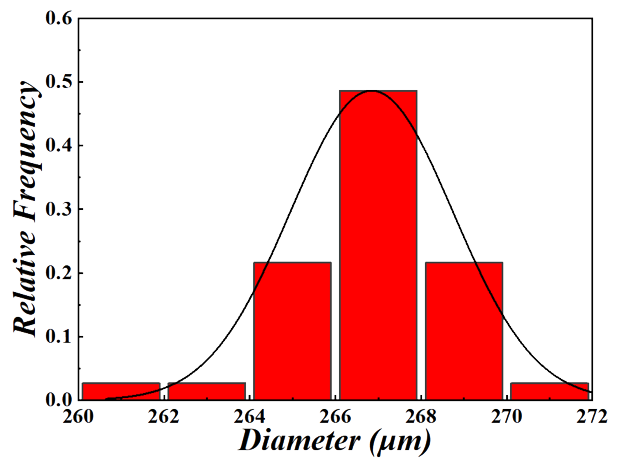
**

**Fig. S1** The particle size distribution map of pollen-inspired PhC barcodes. The average particle size is 266.8 μm; standard deviation is 1.88.

**Figure S2.**

**Fig. S2** Fourier-transform infrared spectroscopy (FTIR) analysis of the pollen-inspired PhC barcodes before and after carboxyl group modification.

**Figure S3.**

**
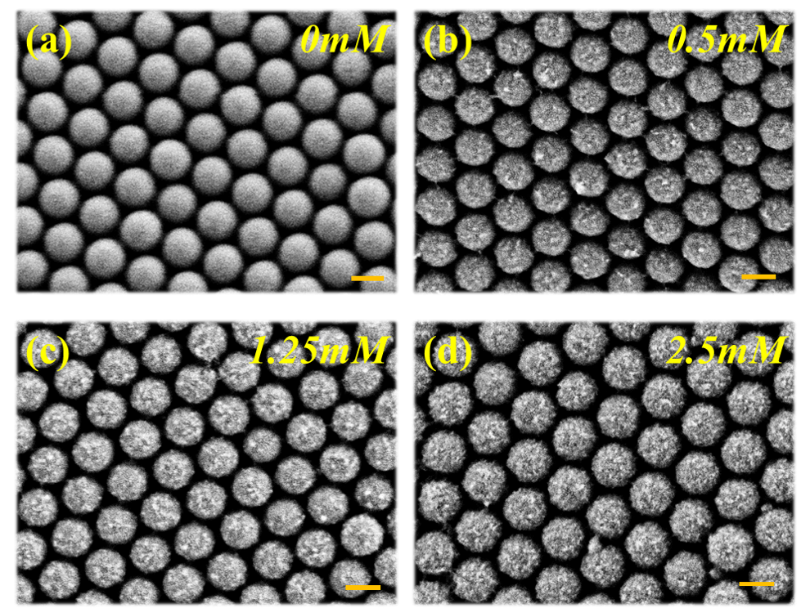
**

**Fig. S3** (a-d) SEM images of the surface of the pollen-inspired PhC barcodes treated with different ion concentrations. Scale bars are 200 nm.

**Figure S4.**

**
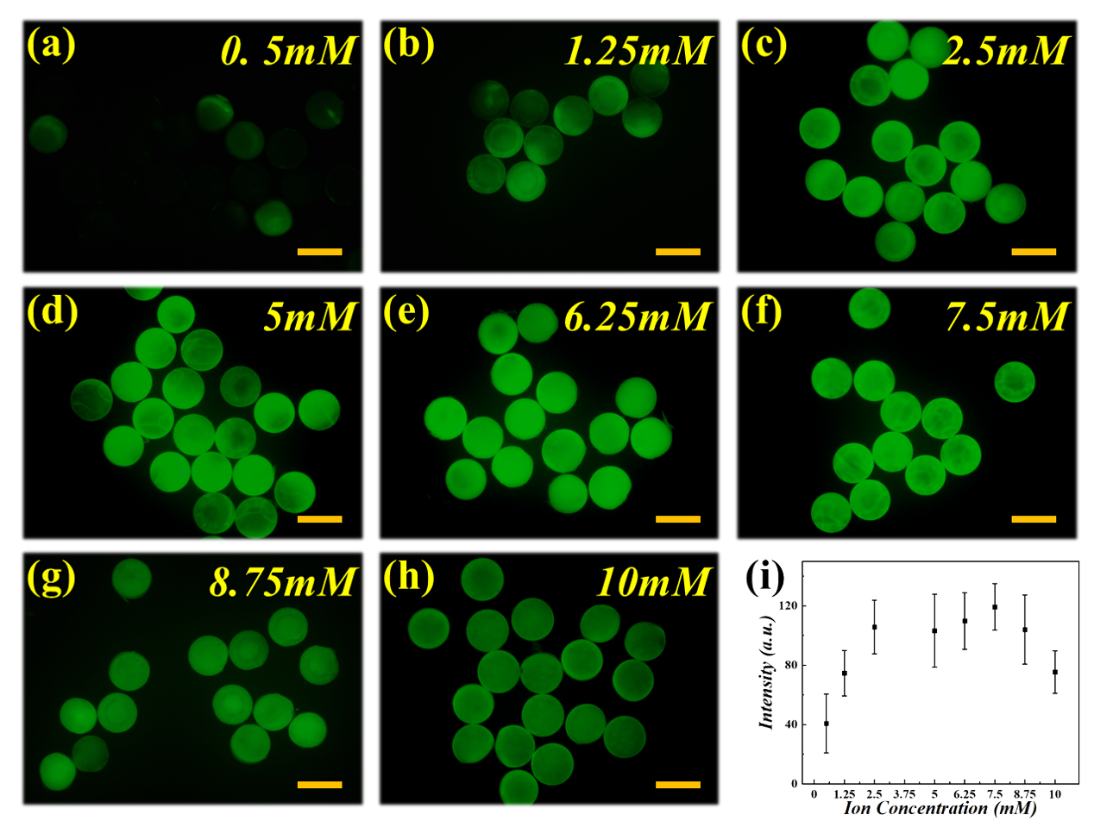
**

**Fig. S4** (a-h) Fluorescence images of pollen-inspired PhC barcodes with different ion concentration after BSA-FITC loading. (i) Optimization curve of the relationship between ion concentration and pollen-inspired PhC barcodes antibody loading ability. Scale bars are 200 μm.

**Figure S5.**

**
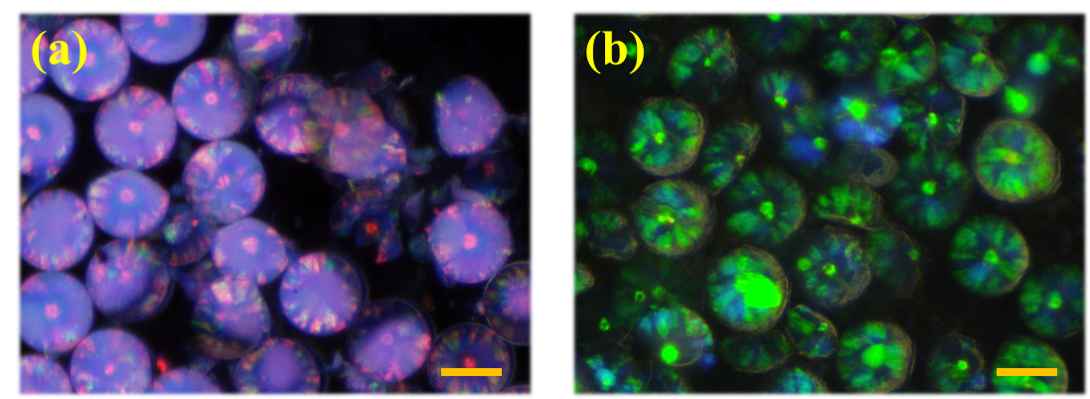
**

**Fig. S5** Optical characterization of the fragmented PhC barcodes due to excessive corrosion. Scale bars are 200μm.

**Figure S6.**

**
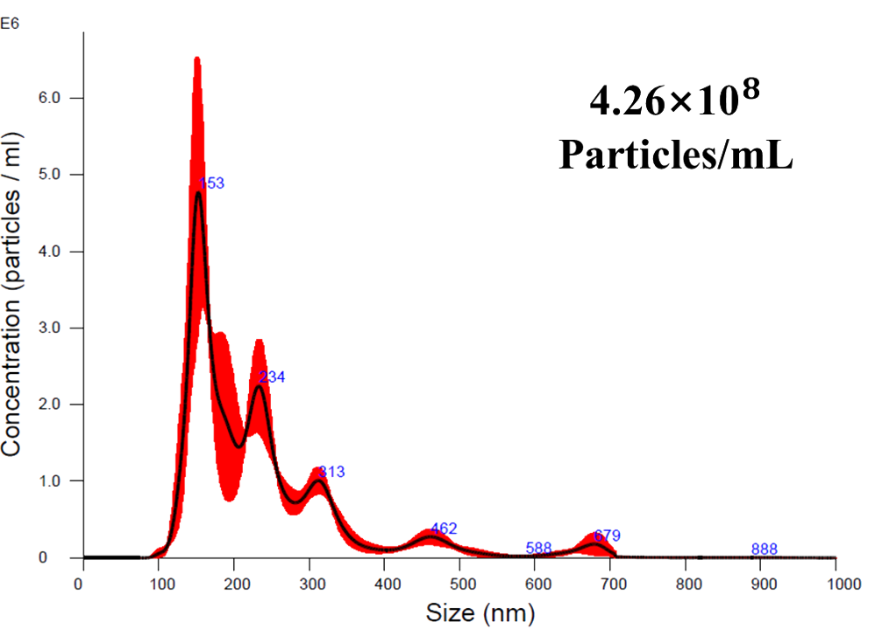
**

**Fig. S6** Nanoparticle tracking analysis (NTA) curve of exosomes sample after diluted 100 times. The sample concentration was 4.26$\text{×}\text{10}^{\text{8}}$ particles/mL.

**Figure S7.**

**
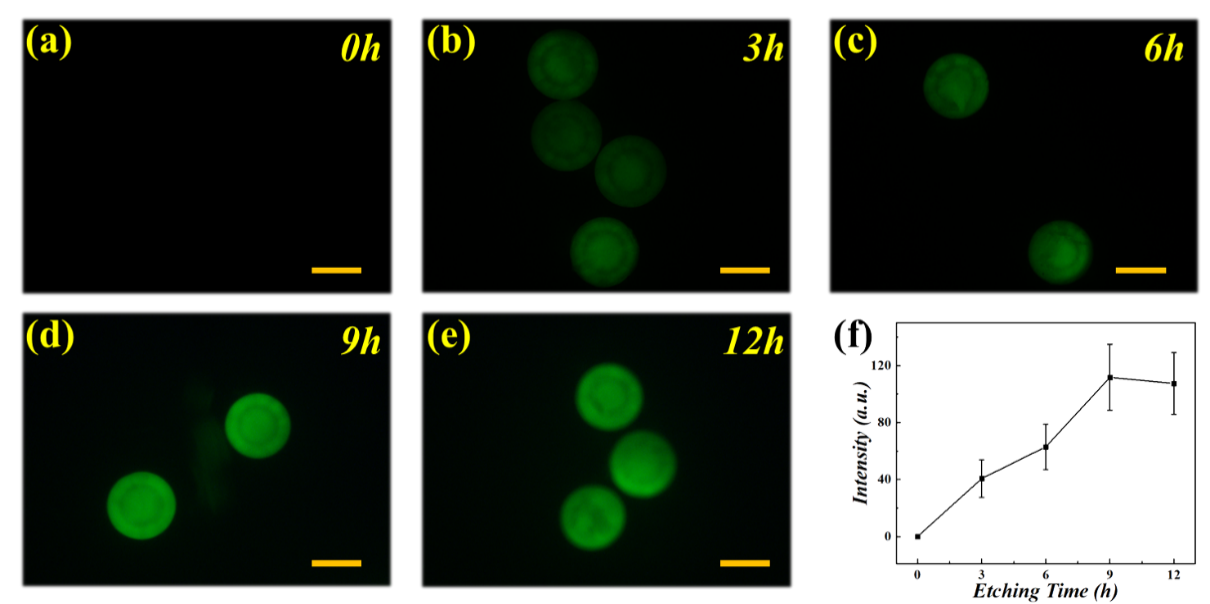
**

**Fig. S7** (a-e) Fluorescence images after blank pollen-inspired PhC barcodes with different etching times (0h, 3h, 6h, 9h, 12h) after exosomes capturing. (f) Curve of the relationship between etching time and fluorescence intensity of blank pollen-inspired PhC barcodes after exosomes capturing. Scale bars are 200 μm.

**Figure S8.**

**
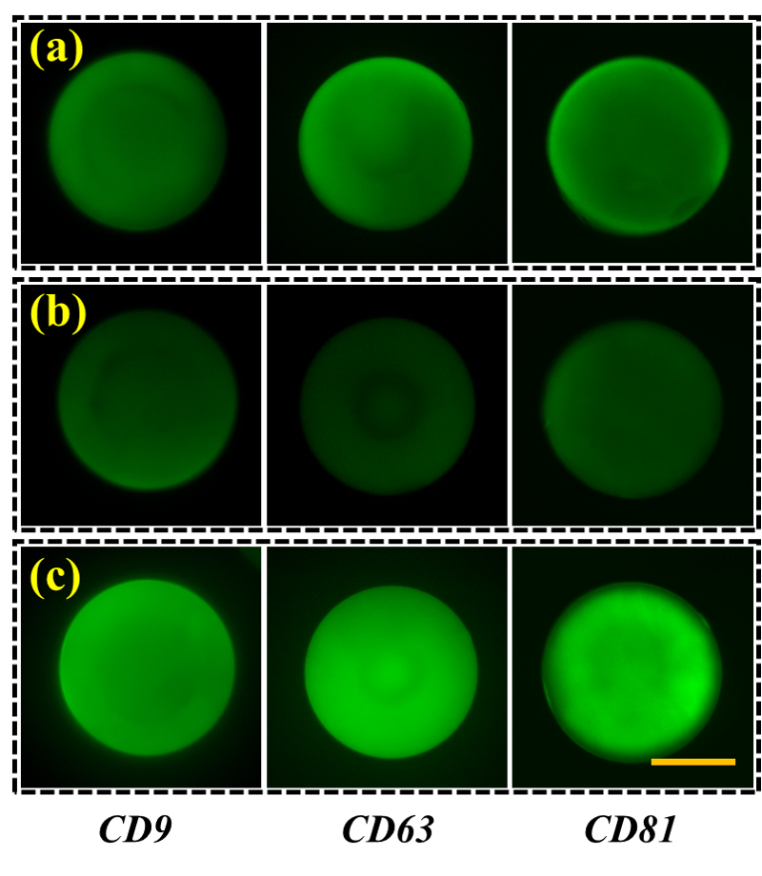
**

**Fig. S8** The fluorescence images of three groups. (a) blank control; (b) BSA blocking; (c) modified with CD9/CD63/CD81 antibodies. Scar bar is 100μm.
